# Progressive multifocal leukoencephalopathy after durvalumab treatment for acute myeloid leukemia: A consequence of an immune reconstitution inflammatory syndrome?

**DOI:** 10.1002/jha2.485

**Published:** 2022-05-29

**Authors:** Emeline Vinatier, Caroline Poli, Aurélien Giltat, Christopher Nunes‐Gomes, Corentin Orvain, Mathilde Hunault‐Berger, Pascale Jeannin, Sylvain Thépot

**Affiliations:** ^1^ CHU Angers Laboratoire d'Immunologie et Allergologie Angers France; ^2^ Univ Angers Nantes Université CHU Angers INSERM CNRS CRCI2NA SFR ICAT Angers France; ^3^ CHU Angers Service des maladies du sang Angers France

**Keywords:** acute leukemia, immunopathology, immunotherapy, infection

## Abstract

Progressive multifocal leukoencephalopathy (PML) is a fatal demyelinating disease of the central nervous system resulting from the reactivation of the John Cunningham virus (JCV). PML occurs almost exclusively during profound immune suppression but it can also be observed in immunocompromised subjects as part of an inflammatory immune reconstitution syndrome (IRIS) in patients receiving antiviral therapy. We report a case of PML in a 61‐year‐old patient with acute myeloid leukemia who had developed after discontinuation of durvalumab (anti‐PD‐L1) therapy initiated after multiple treatments. Results suggest that PML may result from two nonexclusive mechanisms: (i) an inhibition of the protective response of JCV‐specific T cells as a consequence of the blockade of the PD1‐PDL1 pathway, associated with a lack of compensatory expression of other inhibitory receptors by T cells and (ii) a neuroinflammatory response (PML‐IRIS) that may have contributed to virus reactivation.

## 1 INTRODUCTION

Progressive multifocal leukoencephalopathy (PML) is a fatal demyelinating disease of the central nervous system resulting from the reactivation of the John Cunningham virus (JCV). It is estimated that 50%–90% of adults have been exposed to JCV. After an asymptomatic primary infection in the upper respiratory tract, the virus spreads from the primary site of infection to secondary sites including kidneys, lymphoid tissues, and brain to establish latent infection. In the vast majority of cases, the virus remains clinically silent for life, thanks to the presence of protective virus‐specific T cells. PML is a rare complication consecutive to molecular rearrangements of the viral DNA that leads to the production of a pathogenic variant that replicates in glial tissues [1]. PML occurs almost exclusively during profound immune suppression. PML can also occur in immunocompromised subjects during an immune reconstitution inflammatory syndrome (IRIS) reported in patients receiving an antiviral treatment [2].

In September 2017, a 61‐year‐old HIV‐negative woman with an acute myeloid transformation secondary to myelodysplastic syndrome was enrolled in a Phase II clinical trial evaluating the efficacy and safety of durvalumab, a humanized antibody targeting PD‐L1, combined to azacitidine. Treatment consisted in intravenous injection of 1500 mg durvalumab once every four weeks and subcutaneous injection of 75 mg/m^2^ azacitidine for seven days every four weeks. After two cycles, the patient rapidly developed ascending legs paresis and pain. Electromyogram confirmed demyelinating neuropathy, impairment compatible with Guillain–Barre syndrome, as already described after anti‐PD1 Ab treatment [3], and durvalumab treatment was therefore definitely discontinued. Clinical improvement was obtained after few days of corticosteroids and intravenous polyvalent immunoglobulin perfusion. Azacitidine treatment was stopped after eleven cycles in July 2018 because of lack of efficacy (persistence of transfusion dependence; no improvement in blast infiltration of bone marrow).

**FIGURE 1:**
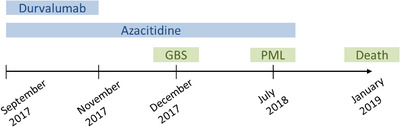
Case report timeline. Therapies appear in blue, clinical events in green. GBS, Guillain–Barre syndrome; PML, progressive multifocal leukoencephalopathy

From July 2018, the patient suffered from a left homonymous hemianopsia. MRI revealed cerebral lesions whose localizations suggested JCV encephalopathy. JCV DNA was detected in cerebrospinal fluid (CSF) by PCR (TaqMan method, JCV large T antigen gene) and lumbar puncture did not found meningitis. Blindness occurred after several months, and the patient died from hematological malignancy progression in January 2019. Timeline is summarized in Figure 1.

## 2 RESULT

**FIGURE 2:**
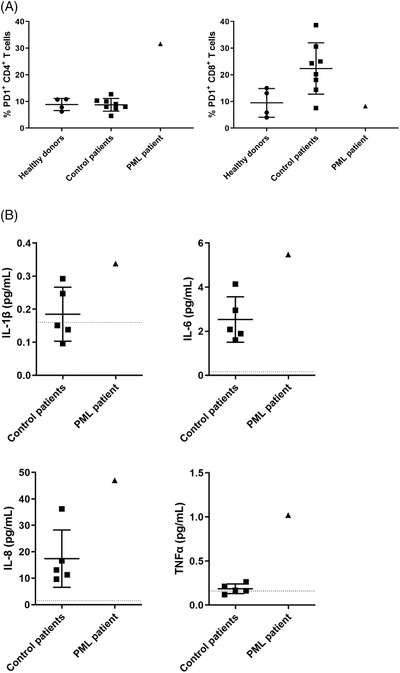
Analysis of the immune status of case report. (A) PD1 expression was analysed by flow cytometry on peripheral CD4+ (left panel) and CD8+ T cells (right panel) from PML patient, healthy donors (circles, *n* = 4) and control patients (squares, *n* = 8). (B) Levels of IL‐1β (upper left panel), IL‐6 (upper right panel), IL‐8 (lower left panel) and TNFα (lower right panel) were determined by Luminex technology in cerebrospinal fluids from PML patient and control patients; dotted line, detection cut‐off. (A, B) Results in healthy subject and control patient groups are expressed as mean ± SEM

Previous studies reported that PML may occur during advanced stages of hematologic malignancies as a result of the immunosuppressive nature of the disease and/or treatment (cytotoxic drugs, stem cell transplantation, total body irradiation or immunomodulatory molecules) [4, 5]. We thus hypothesized that discontinuation of durvalumab may have induced reduced functions of JCV‐specific T cells consecutive to a rebound increase of inhibitory immune checkpoint expression. We then analyzed the expression of inhibitory coreceptors on peripheral T cells in the PML patient, in healthy donors (*n* = 4) and in myelodysplastic patients treated with hypomethylating agents (control patients, *n* = 8), as these drugs may enhance the expression of immune checkpoints [6]. Results showed that PD1 expression was markedly increased on peripheral CD4+ T cells of the PML patient compared to healthy donors and control patients (Figure 2A). Conversely, PD1 expression on CD8+ T cells of the PML patient was equivalent to healthy donors and reduced compared to control patients (Figure 2A). We also analyzed the expression of the coinhibitory molecules CTLA‐4, BTLA, LAG3, 2B4, TIM3, CD160, and TIGIT to evaluate a possible compensatory overexpression of other inhibitory receptors. Results showed that levels of these molecules on CD4+ and CD8+ T cells of the patient were equivalent to healthy donors (supplementary data).

We then explored whether PML may be related to IRIS. Indeed, the patient developed Guillain–Barre Syndrome (GBS), controlled with corticosteroids and intravenous immunoglobulins, 2 months after the initiation of durvalumab. This acute inflammatory demyelinating polyneuropathy is usually considered as an immune‐related adverse event under immune checkpoint inhibitors that results from an overactivation of the immune system and leading to massive inflammation. As previously described in GBS [7], the inflammatory cytokines IL‐1β, IL‐6, IL‐8, and TNFα were increased in CSF of the PML patient, compared to control patients (*n* = 5, Figure 2B).

## 3 DISCUSSION

In this study, we report a case of PML in a patient with acute myeloid leukemia. PML is a challenging disorder to manage. Elucidating the underlying mechanisms and aggravating factors is thus of great importance. Our results suggest that PML may result from two nonexclusive mechanisms: (i) the failure of JCV‐specific T‐cell response consecutive to the inhibition of PD1‐PDL1 pathway, as no compensatory overexpression of other inhibitory receptors on patient T cells was observed and (ii) a neuroinflammation (PML‐IRIS) that may have led to virus reactivation and replication.

PML prognostic is very poor as demyelination and neuronal death are fatal within 2–15 months after diagnosis and often faster in patients with hematologic malignancies. The only therapies able to stabilize or even improve PML symptoms are anti‐PD1 or anti‐PDL1 Abs [8, 9] and adoptive T‐cell therapy [2]. In vitro studies reported that blocking PD1 increases IFNγ production by JCV‐specific CD8+ T cell in PML patients. Moreover, the expression of PD1 is increased on PML T cells compared to healthy subjects [10]. Our results evidence an increase of PD1 expression only on CD4+ T cells, partially consistent with literature. This overexpression of PD1 on CD4+ T cells sustains the hypothesis that specific T responses could no longer counteract JCV spreading after durvalumab arrest. It is noteworthy that the expression of checkpoint inhibitors by peripheral blood T cells may be different to brain‐resident cells and may constitute a limit of our description.

IRIS mainly occurs in response to newly acquired opportunistic pathogens (unmasking IRIS) or to previously acquired opportunistic infections, paradoxically aggravating their symptoms (paradoxical IRIS) [2]. As an example, the restoration of T‐cell functions induced by immune checkpoint inhibitors can reactivate latent tuberculosis and acute progression of aspergillosis [11]. We thus hypothesized that IRIS could be also involved in the reactivation of JCV. Indeed, the transduction pathways downstream inflammatory cytokine receptors regulate the expression of genes involved in JCV replication and neurovirulence [12]. This viral flare‐up could not be contained by specific T cells in immunosuppressed patients, leading to neuropathogenic JCV spreading and, consequently, PML lesions. IRIS frequently occurs within 4 to 8 weeks after antiviral treatment in HIV patients. In our case, inflammatory cytokines were detected in CSF 2 months after initiation of durvalumab, which is consistent with what is observed in HIV patients [2].

In our case, PML occurred 8 months after durvalumab arrest. This timing is compatible with the course of infection which occurs at least 6 months before clinical manifestations of PML [13, 14], making it difficult to attribute one treatment or another to the onset of the disease. Nevertheless, IRIS depends on the timing of immune restoration [2] and one can thus hypothesize that abrupt durvalumab discontinuation may promote PML‐IRIS. Nevertheless, in the absence of consensus criteria for non‐HIV IRIS [15], this entity is hard to diagnose. IRIS‐mediated JCV reactivation has been already suspected under pembrolizumab and nivolumab [11], strengthening the possibility of PML‐IRIS being a consequence of treatment with immune checkpoint inhibitors in some patients.

In conclusion, we describe the first case of PML occurring after discontinuation of durvalumab therapy in an immunocompromised patient treated with hypomethylating agent who had undergone multiple treatments prior to durvalumab therapy. This observation suggests that attempting to restore T‐cell immunity in a very advanced cancer may be insufficient to restore effective viral‐immunity and may induce deleterious inflammation leading to IRIS‐associated infections. Further observations of unusual cases like the one reported here are needed to decipher the pathophysiological process associated with PML in patients treated with immune checkpoint inhibitors.

## AUTHOR CONTRIBUTIONS

MHB, PJ, and ST supervised the project. EV, CP, and AG performed research. EV, CP, AG, CNG, and CO performed data analysis. EV, CP, and AG wrote the report. CNG, CO, MHB, PJ, and ST provided critical revision of the manuscript. All authors read and approved the submitted version.

## CONFLICT OF INTEREST

The authors declare that the research was conducted in the absence of any commercial or financial relationships that could be construed as a potential conflict of interest.

## Supporting information



Supporting Information
