# Supplementary material for: Progressive multifocal leukoencephalopathy after durvalumab treatment for acute myeloid leukemia: A consequence of an immune reconstitution inflammatory syndrome?
Source: EJHaem. 2022 May 29;3(3):958–61. doi: 10.1002/jha2.485 (PMC9422031; doi:10.1002/jha2.485)
Supplement: Supplementary file 1 — Supporting Information [file JHA2-3-958-s001.pdf]

Supplementary data: Analysis of coinhibitory and costimulatory molecules on CD4+ (A) and CD8+(B) T cells. Coinhibitory proteins CTLA-4, CD160, BTLA, LAG3, 2B4, Tim3, PD-1, TIGIT and costimulatory proteins CD96, CD28, LIGHT expression was analyzed by flow cytometry on CD4+ T cells (CD3+ CD4+ CD8-) and CD8+ T cells (CD3+ CD4- CD8+) from the PML patient and from control patients (CP 1-8), and healthy donors (HD 1-4). Results are shown as histogram (grey: isotype control, red : specific Ab).

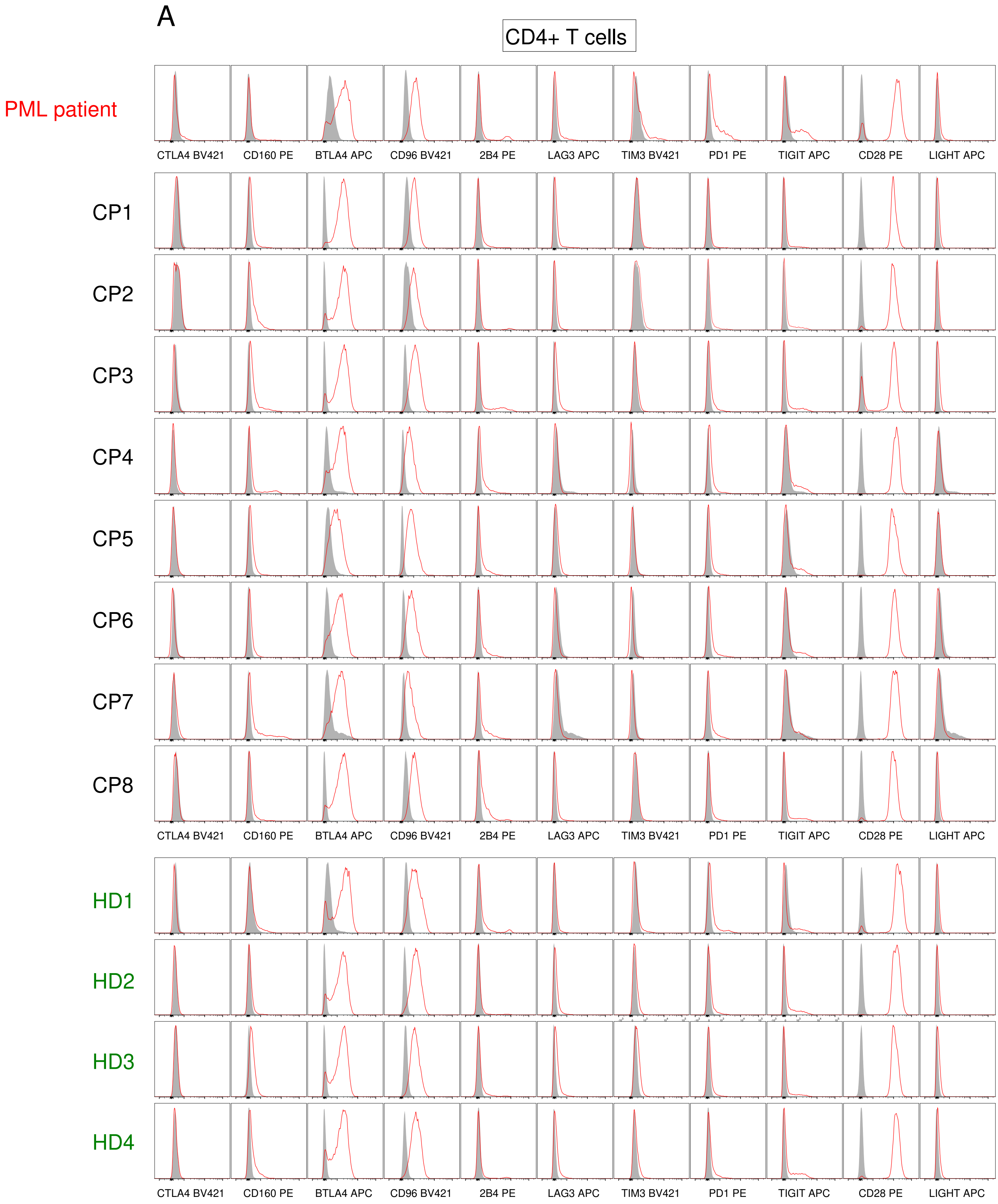
A
CD4+ T cells
PML patient
CP1
CP2
CP3
CP4
CP5
CP6
CP7
CP8
HD1
HD2
HD3
HD4
CTLA4 BV421
CD160 PE
BTLA4 APC
CD96 BV421
2B4 PE
LAG3 APC
TIM3 BV421
PD1 PE
TIGIT APC
CD28 PE
LIGHT APC

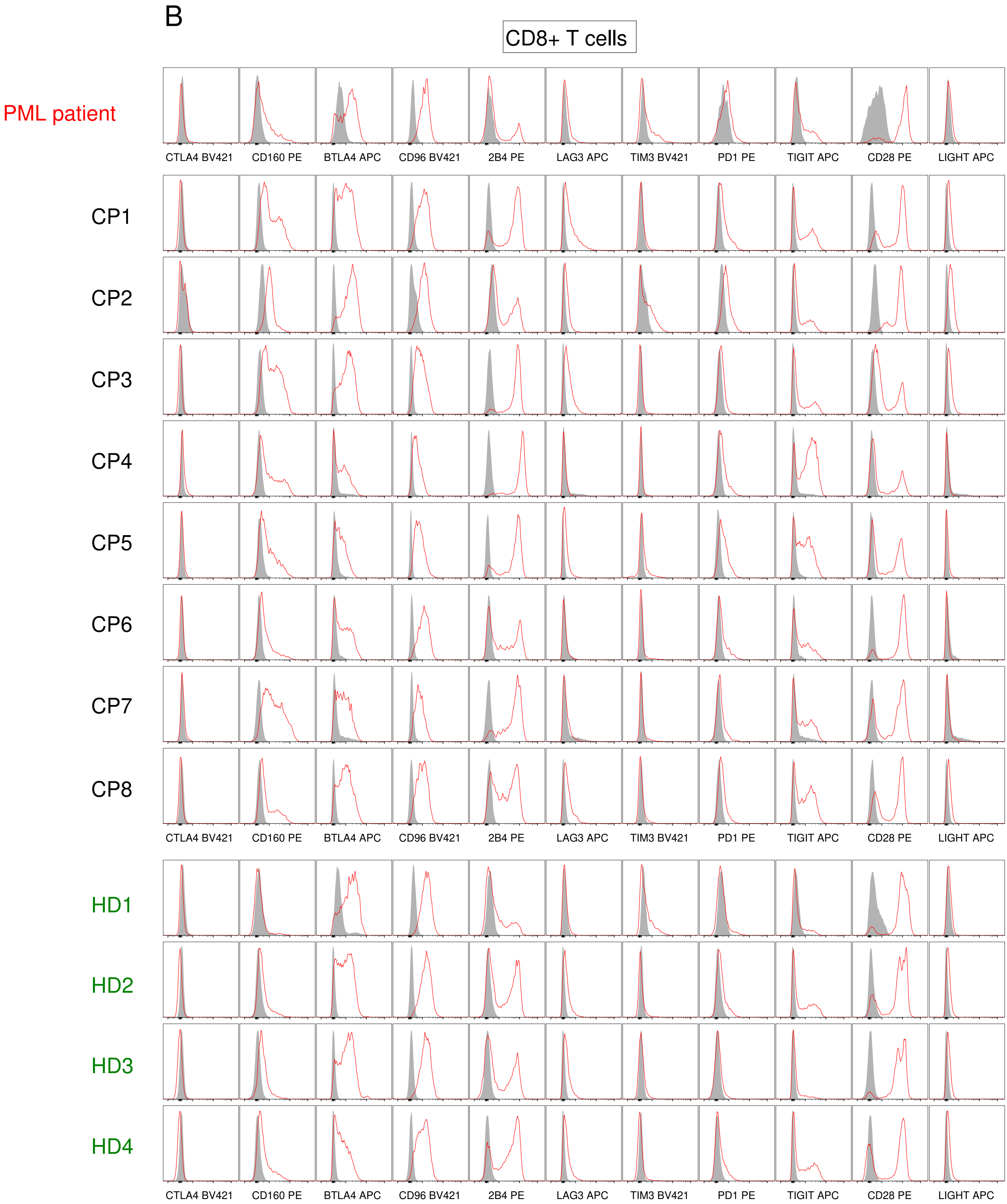

B
CD8+ T cells
PML patient
CP1
CP2
CP3
CP4
CP5
CP6
CP7
CP8
HD1
HD2
HD3
HD4
CTLA4 BV421
CD160 PE
BTLA4 APC
CD96 BV421
2B4 PE
LAG3 APC
TIM3 BV421
PD1 PE
TIGIT APC
CD28 PE
LIGHT APC
